# Thymoquinone’s Antiviral Effects: It is Time to be Proven in the Covid-19 Pandemic Era and its Omicron Variant Surge

**DOI:** 10.3389/fphar.2022.848676

**Published:** 2022-04-05

**Authors:** Maen Abdelrahim, Abdullah Esmail, Noor Al Saadi, Eva Zsigmond, Ebtesam Al Najjar, Doaa Bugazia, Hadeel Al-Rawi, Ayat Alsaadi, Ahmed O. Kaseb

**Affiliations:** ^1^ Houston Methodist Cancer Center, Houston Methodist Hospital, Houston, TX, United States; ^2^ Cockrell Center for Advanced Therapeutic Phase I Program, Houston Methodist Research Institute, Houston, TX, United States; ^3^ Weill Cornell Medical College, Institute of Academic Medicine, Houston, TX, United States; ^4^ Houston Methodist Research Institute, Houston, TX, United States; ^5^ Faculty of Medicine and Health Sciences, University of Science and Technology, Sanaa, Yemen; ^6^ Faculty of Medicine, Xavier University School of Medicine Aruba, Oranjestad, Aruba; ^7^ Faculty of Medicine, University of Tripoli, Tripoli, Libya; ^8^ Faculty of Medicine, University of Jordan, Amman, Jordan; ^9^ Department of Biology and Chemistry, Buffalo State College, Buffalo, NY, United States; ^10^ Department of Medical Oncology, The University of Texas MD Anderson Cancer Center, Houston, TX, United States

**Keywords:** COVID-19, pandemic, Coronavirus, Thymoquinone, PAXLOVID, molnupiravir, COVID-19 vaccines and anti-viral agents, Omicron variant

## Abstract

The COVID-19 pandemic has impacted every country in the world. With more than 400 million cases and more than 5.5 million deaths. The FDA either approved or authorized the emergency use for three vaccines against COVID-19. The treatment options of COVID-19 are very limited. Multiple complementary and alternative medicine modalities were suggested to be efficacious in the treatment of COVID-19 such as Thymoquinone. The effects of Thymoquinone have been examined and multiple studies indicate a promising beneficial effect. However, the current body of research is limited in terms of its scope, quality, and quantity. While higher-quality studies are required, physicians do not routinely recommend the use of marketed supplements of natural products, including Thymoquinone for COVID-19. Given the numerous suggested positive effects of Thymoquinone, including anti-inflammatory and antimicrobial properties, additional research is required to confirm or refute these promising benefits. Complementary and alternative medicine is an area that requires additional evidence-based practice and research to confirm effects observed in clinical practice.

## Introduction

The COVID-19 pandemic has impacted almost every country in the world. With more than 400 million cases and around 5.5 million deaths, finding a treatment is a priority (World Health Organization, 2021a). However, the necessity of finding a treatment has led to the adoption of non-evidence-based practices. Hydroxychloroquine was one of the first medications to be proposed as a possible treatment for COVID-19. Additionally, multiple complementary and alternative medicine strategies have been suggested as possible treatments of COVID-19 (Ang et al., 2020; Badakhsh et al., 2021).

In addition to the recently discovered COVID-19, six unique strains of human coronaviruses have been identified (Elfiky, 2020; Hui et al., 2020). Coronaviruses are around 30 kb enclosed, positive-sense single-stranded RNA viruses. They infect a wide variety of hosts (Channappanavar et al., 2014). Coronaviruses are classified into four genera based on their genetic structure: α, β, γ, and δ. Only mammals are infected by the α and β coronaviruses (Rabi et al., 2020). The common cold and croup are caused by human coronaviruses such as 229E and NL63, which belong to the alpha coronavirus family. β Coronaviruses, on the other hand, include SARS-CoV, OC43, Middle East respiratory syndrome coronavirus (MERS-CoV), and SARS-CoV-2 are the most dangerous and are responsible for roughly 800 fatalities each year. According to the WHO, SARS HCoV has a 10% fatality rate, whereas MERS HCoV has a 36% mortality rate (World Health Organization, 2021b).

Since the emergence of COVID, physicians and researchers have struggled to effectively treat the novel coronavirus. More recently medications of varying levels of effectivity have been implicated in the treatment of COVID 19. These include steroids, antiviral drug Remdesivir (RDV), and monoclonal antibody (mAb) (Beigel et al., 2020; Deb et al., 2021). The mAbs are thought to help reduce the viral load by blocking virus entrance into cells by binding to viral spikes and therefore preventing virus attachment to cell surface receptors (Deb et al., 2021). The mAbs may potentially target host cell receptors or co-receptors, rendering the host cells’ binding sites inaccessible to SARS-CoV-2. Alternatively, mAbs can act as immunosuppressive agents, limiting immune-mediated damage (Deb et al., 2021). Most recently, *Molnupiravir* as early treatment has shown a reduced risk of hospitalization or death in at-risk, unvaccinated adults with Covid-19 (Jayk Bernal et al., 2021). furthermore, *Pfizer* released phase 2/3 results from the *PAXLOVID* trial, confirming the novel COVID-19 oral antiviral treatment’s robust efficacy in reducing the risk of hospitalization or death by 89% (within 3 days of symptom onset) and 88% (within 5 days of symptom onset) compared to placebo; no deaths in non-hospitalized, high-risk adults with COVID-19 compared to placebo (Archive, 2021), in addition, *PAXLOVID* has been authorized by the United States Food and Drug Administration (Ashraf et al., 2021) as emergency use authorization and became the first oral antiviral authorized by FDA for treatment of COVID-19 (U.S. Food and Drug Administration, 2021a).

Three COVID-19 vaccines, *Pfizer-BioNTech*, *Moderna*, and *Johnson & Johnson’s Janssen*, were either approved or authorized for emergency use by the FDA (U.S. Food and Drug Administration, 2021b; U.S. Food and Drug Administration, 2021c; U.S. Food and Drug Administration, 2021d). The vaccines were effective in 90% of people regardless of age, gender, and underlying health issues. Furthermore, effectiveness was demonstrated in a subsequent analysis that included people with and without evidence of past SARS-CoV-2 infections (Oliver et al., 2020). Discomfort at the injection site, muscle pain, chills, joint pain, fatigue, headache, and fever were described as common adverse events of the COVID19 vaccinations (Centers for Disease Control and Prevention, 2021a; Abdelrahim and Esmail, 2021). Adverse effects following the second dose included pain at the injection site, muscle pain, chills, joint pain, tiredness, headache, and fever often lasted several days more than the first dose (Abdelrahim and Esmail, 2021; U.S. Food and Drug Administration, 2021e).

**FIGURE 1:**
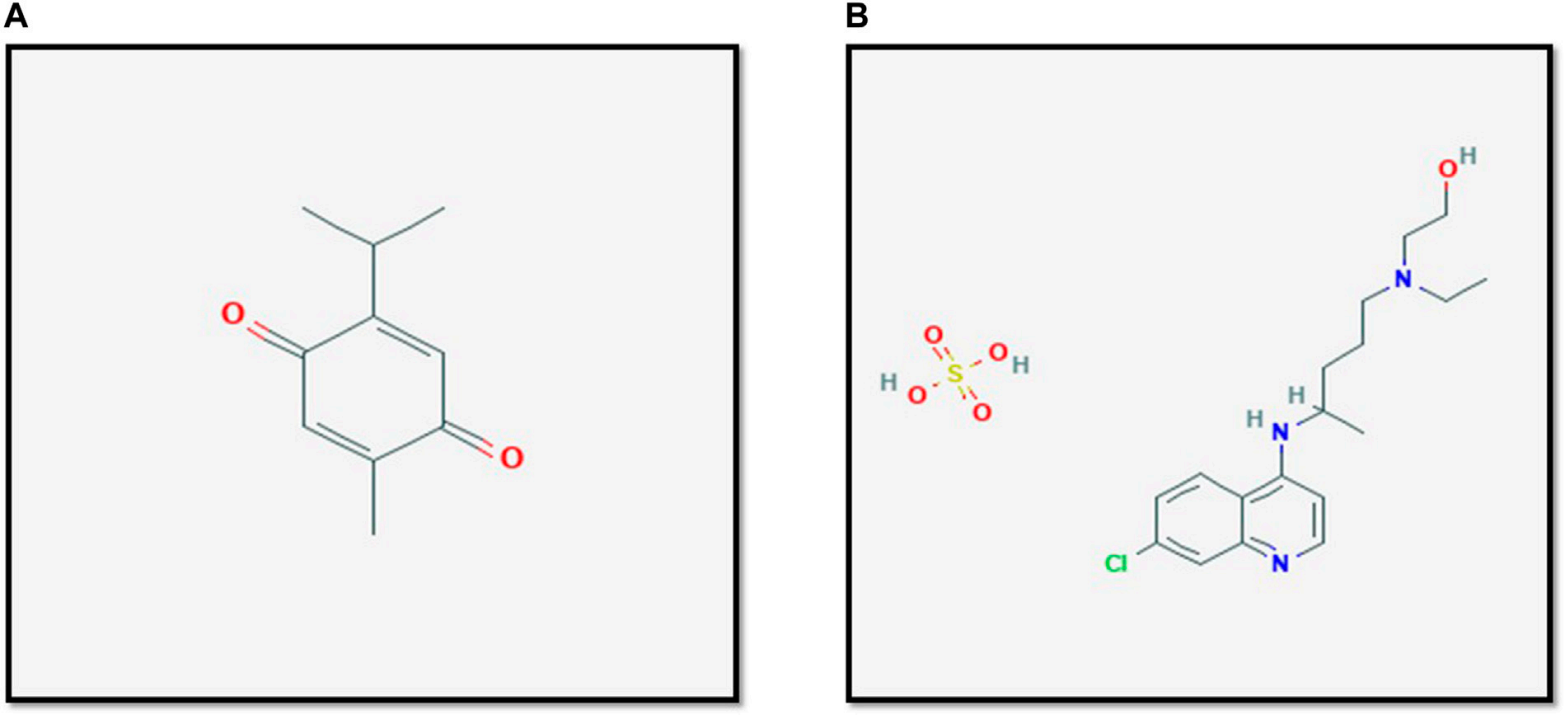
The structure of Thymoqunione **(A)** and Hydroxychloroquine **(B)**. The molecular formula of Hydroxychloroquine sulfate is C_18_H_28_ClN_3_O_5_S, whereas it is C_10_H_12_O_2_ for Thymoquinone. Thus, it is unlikely for both chemical structures to have similar effects. Figures are adapted from PubChem (National. Center for Biotechnology Information, 2021a; National. Center for Biotechnology Information, 2021b).

Multiple complementary and alternative medicine modalities where there suggested to be efficacious in the treatment of COVID-19. Suggested treatment options in the scientific literature include *Thymoquinone* and its natural source *Nigella sativa* (Bouchentouf and Noureddine, 2020). *Thymoquinone* is a component of many plants, with *Nigella sativa* being its primary natural source. *Nigella sativa* is used complementary or alternative medicine for its many proposed effects by different cultures and traditions (Goyal et al., 2017). The molecular formula of *Thymoquinone* is C_10_H_12_O_2_, whereas it is C_18_H_28_ClN_3_O_5_S for *Hydroxychloroquine sulfate*. Thus, it is unlikely for both chemical structures to have similar effects Figure1. Table 1 shows the different plants that contain *Thymoquinone. Thymoquinone* was found to have possible effects on certain biological functions (Khader and Eckl, 2014) and this led to some interest in studying the anti-microbial properties of *Thymoquinone* (Forouzanfar et al., 2014). Studies on the anti-viral effects of *Thymoquinone* are limited in literature; however, multiple *in-vitro* and *in-vivo* studies suggest some therapeutic potential. Moreover, Salim and Nour (Bouchentouf and Noureddine, 2020) have recently demonstrated that compounds other than *Thymoquinone* within the *Nigella sativa* plant may play a role in targeting COVID-19.

**TABLE 1:** Plants containing *Thymoquinone*.

Plants Containing *Thymoquinone*
*Nigella sativa*
*Satureja Hortenis*
*Eupatorium Cannabinum*
*Juniperus Communis*
*Monarda Didyma*
*Monarda Media*
*Monarda Menthifolia*
*Thymus Pilegioides*
*Thymus Serpyllum*
*Thymus Vulgaris*
*Urejamontana*

This review is a timely review of *Thymoquinone’s* properties as an anti-viral agent. Similar reports are needed to keep the medical community updated regarding the efficacy of various alternative medicines so that medical professionals can inform and educate patients. This review aims to illustrate the role *Thymoquinone* effect in the immunological response to COVID-19 and other viral infections. In addition, we hope to shed the light on the potential drug development and the clinical utility of *Thymoquinone* to treat COVID-19 patients which is an era of unmet need for the time being.

## Coronavirus Overview

In the downstream areas of Open Reading Frame 1 (ORF 1), all coronaviruses have particular genes that encode proteins for viral replication, nucleocapsid development, and spike creation (Elfiky, 2020). The glycoprotein spikes on coronaviruses’ outer surface are essential for the virus’s attachment and penetration into host cells. The MERS-coronavirus requires dipeptidyl peptidase 4 (DPP4), whereas the HCoV-NL63 and SARS-coronaviruses require angiotensin-converting enzyme 2 (ACE2) as a major receptor (Gralinski and Menachery, 2020). The cell-surface Heat Shock Protein A5 (HSPA5), also known as GRP78 or BiP, has been found to be identified by the viral spike proteins of SARS-Cov-2 (Datau et al., 2010). SARS-CoV-2 employs the same ACE2 cell receptor and method for entrance into host cells as SARS-CoV, these details were validated in a fluorescence experiments (Gralinski and Menachery, 2020; Shereen et al., 2020; Xu et al., 2020).

Attachment, penetration, biosynthesis, maturity, and release are the five phases in a virus’s life cycle within the host. Viruses enter host cells by endocytosis or membrane fusion after binding to host receptors (attachment) (penetration). The components of the virus are subsequently released into the host cells, and viral RNA is taken into the nucleus for replication. Viral proteins are made from viral mRNA (biosynthesis). Finally, new virus particles (maturation) are produced and discharged. Spike (S), membrane (M), envelop (E), and nucleocapsid (N) are the four structural proteins found in Coronaviruses (N) (Walls et al., 2020). The spike protein is a transmembrane trimetric glycoprotein that protrudes from the viral surface and controls coronavirus diversification and host tropism. Spike proteins are made up of two functional subunits: the S1 subunit is in charge of binding to the host cell receptor, and the S2 subunit is in charge of fusing the viral and cellular membranes. The ACE2 receptor has already been identified as a functioning SARS-CoV receptor (Li et al., 2003). The spike protein for SARS-CoV-2 interacts with ACE2 according to structural and functional investigations (Chen et al., 2020; Letko et al., 2020; Walls et al., 2020). ACE2 is highly expressed in the lungs, heart, ileum, kidneys, and bladder (Zou et al., 2020). ACE2 is a highly expressed epithelial cell of the lungs. Following SARS-attachment CoV-2’s to the host protein, the spike protein is cleaved by proteases. The S1 and S2 subunits remain non-covalently linked after cleavage at the S1/S2 cleavage site, and the distal S1 subunit aids in the prefusion stabilization of the membrane-anchored S2 subunit (Walls et al., 2020). Following cleavage at the S2 site, the spike protein is probably activated for membrane fusion with irreversible conformational changes.

Antigen presentation by dendritic cells (DCs) and macrophages trigger T cell responses against coronaviruses. DCs and macrophages can phagocytize virus-infected apoptotic cells (Fujimoto et al., 2000). DCs and macrophages, for example, can phagocytize virus-infected apoptotic epithelial cells, resulting in antigen presentation to T cells. In addition to ACE2, SARS-CoV may bind to dendritic cell-specific intercellular adhesion molecule-3-grabbing nonintegrin (DC-SIGN) and DC-SIGNR (L-SIGN) and DC-SIGNR-related protein (DC-SIGNR, L-SIGN) (Jeffers et al., 2004; Marzi et al., 2004; Yang et al., 2004). Dendritic cells and macrophages both express high numbers of DC-SIGN. To present viral antigens to T cells, these antigen-presenting cells go to the draining lymph nodes. T lymphocytes, both CD4^+^ and CD8^+^, play an important function. CD4^+^ T cells stimulate B cells to produce virus-specific antibodies, whereas CD8^+^ T cells destroy virally infected cells. Regarding to coronavirus variants, the scientists track all variants, however, some are classified as variants to be monitored, variants of interest, variants of high consequence, and variants of concern such as *Omicron*–B.1.1.29 and *Delta*–B.1.617.2 (Centers for Disease Control and Prevention, 2021b; Karim and Karim, 2021). Some variants spread more easily and quickly than others, for example, the *Omicron* variant may spread more easily than other variants, including Delta (Centers for Disease Control and Prevention, 2021a). These classifications are based on the ease with which the variant spreads, the severity of the symptoms, how well the variant responds to treatments, and how well immunizations protect against the variant.

## Scientific Report

Several synthetic compounds initially thought to have shown promise in COVID-19 therapy, including hydroxychloroquine and chloroquine phosphate (Cortegiani et al., 2020; Gao et al., 2020) and newer antiviral drugs like lopinavir (Yao et al., 2020), have subsequently been shown to have little or no effect on hospitalized COVID- 19 patients, as indicated by overall mortality, initiation of ventilation and duration of hospital stay (Pan et al., 2021). On the other hand, *Remdesivir* (Holshue et al., 2020; Wang et al., 2020) clinical data suggest efficacy in treating COVID-19 and is the first FDA-approved COVID-19 therapy (Lamb, 2020).

The creation of innovative antiviral medications may be driven by traditional herbal medicines and purified natural ingredients. For example, Emetine an isoquinoline alkaloid isolated from *Cephaelis ipecacuanha* is an effective amoebicidal drug. Similarly, the drug quinine is derived from *Cinchona* tree bark. Other common drugs derived from natural compounds include *aspirin*, *morphine*, and *paclitaxel*, an antineoplastic drug (Ganjhu et al., 2015). Between 1981 and 2014, half of all medications approved were derived from or resembled a natural component (Newman and Cragg, 2016).

According to scientific investigations, *Nigella sativa* (Family Ranunculaceae) is developing as a therapeutic plant with a wide range of pharmacological potential. *Nigella sativa*, often known as black seed, is native to Southern Europe, North Africa, and Southwest Asia. It is also cultivated in other regions of the world, including the Eastern Mediterranean and India (Khare, 2004). *Nigella sativa* is a commonly used medicinal herb in several traditional medical systems across the world, including Unani and Tibb, Ayurveda, and Siddha. The seeds and oil of the plant have a long history of use as both medicinal and sustenance (Ahmad et al., 2013). The star of this study, *Thymoquinone* is one such product derived from *Nigella sativa*. *Thymoquinone* has been investigated for its potential anti-inflammatory, anti-microbial, and anti-tumor effects (Beigel et al., 2020) (Bouchentouf and Noureddine, 2020). Most of these studies have been performed *in vitro* or animal-based models. However, very few studies have been able to establish clear clinical evidence of therapeutic effects.

Experiments demonstrate that *Thymoquinone* inhibits the growth of a variety of bacteria. Different extracts of *Nigella sativa* showed possible effects on multiple bacteria, including extracts that contained *Thymoquinone* alone. Chaieb et al. (2011) showed that *Thymoquinone* was effective against seven out of sixteen tested bacteria. These bacteria were mainly Gram-positive bacteria. Other studies have confirmed that *Thymoquinone* has the most potent effect on Gram-positive bacteria (Kokoska et al., 2008). Additionally, *in-vivo* studies using animal models have also suggested possible positive effects. In an acute pyelonephritis model, treatment with *Thymoquinone* (a dose of 10 mg/kg) was given before bacterial inoculation of E-Coli and *Thymoquinone* was also repeated every 24 h. Histological examination exhibited a reduction in oxidative damage and a nephron-protective effect due to *Thymoquinone* treatment (Evirgen et al., 2011).

The effects of *Thymoquinone* have also been studied in fungal infections. Both *in vitro* and *in vivo* studies have suggested that *Thymoquinone* may play a possible therapeutic role in the treatment of different fungal infections, such as dermatophytes and *candida* (Aljabre et al., 2005). Like in anti-bacterial studies, anti-fungal experiments also have inconsistencies in dosing, the type of extracts, as well as a lack of clinical proof.

In direction of antiviral effect, *Thymoquinone’s* antiviral effectiveness against various viral infections has been supported by several studies, according to its multiple positive benefits, including antioxidant, anti-inflammatory, and immunomodulatory properties, as well as the possibility of direct viral elimination, and these studies reported that the viral loads in the liver and spleen were dramatically reduced, which correlated with increased IFN- production and CD4 (+) T cell response (Forouzanfar et al., 2014; Sommer et al., 2020; Salem and Hossain, 2000). In addition, multiple studies have suggested the same concept of that *Thymoquinone* has antiviral properties specifically in regards to cytomegalovirus (CMV), human immunodeficiency virus (Archive, 2021), and influenza. In contrast to other anti-microbial studies, these reports included clinical outcomes which highlight the possible efficacy of *Thymoquinone* as a therapeutic agent for HIV (Onifade et al., 2015). Two patients who were ineligible for the highly active antiretroviral therapy (HAART) achieved seroconversion with *Thymoquinone*. One of the patients was a 27-year-old pregnant female, who achieved seroconversion and no vertical transmission. In another study, *Thymoquinone* was shown to decrease hyperinsulinemia associated with HAART therapy (Chandra et al., 2009).

These promising studies support the need for further investigation. Table 2 summarizes some selected examples of the antiviral effects of *Thymoquinone.*

**TABLE 2:** Selected examples of the anti-viral effects of *Thymoquinone* (TQ) and *Nigella sativa* extracts.

Virus	Type of study	Comments and outcomes	References
CMV	Animal	- Study has been done using Murine CMV	Salem and Hossain, (2000)
		- Possible *in-vitro* effect in inhibition of CMV	
		- Increase in interferon-gamma and macrophages number	
Human immunodeficiency virus	Case reports	- 27-year-old pregnant female ineligible for HAART, achieved seroconversion and no vertical transmission	Onifade et al. (2015)
Human immunodeficiency virus	Animal	- Decrease in HAART-related hyperinsulinemia in treated rats	Chandra et al. (2009)

In addition to *Thymoquinone,* anti-viral effects of compounds found in the oils of the *Nigella sativa* plant have also been examined, as illustrated by *in-vitro* and animal studies of the murine CMV (Salem and Hossain, 2000). *Thymoquinone* treatment with or without curcumin led to reduced symptoms and viral shedding in animals infected by the H9N2 virus, a form of avian influenza that affects poultry and in more rare cases humans (Cortegiani et al., 2020). Other extracts have been shown to have probable effects in the treatment of hepatitis C (Barakat et al., 2013).

**FIGURE 2:**
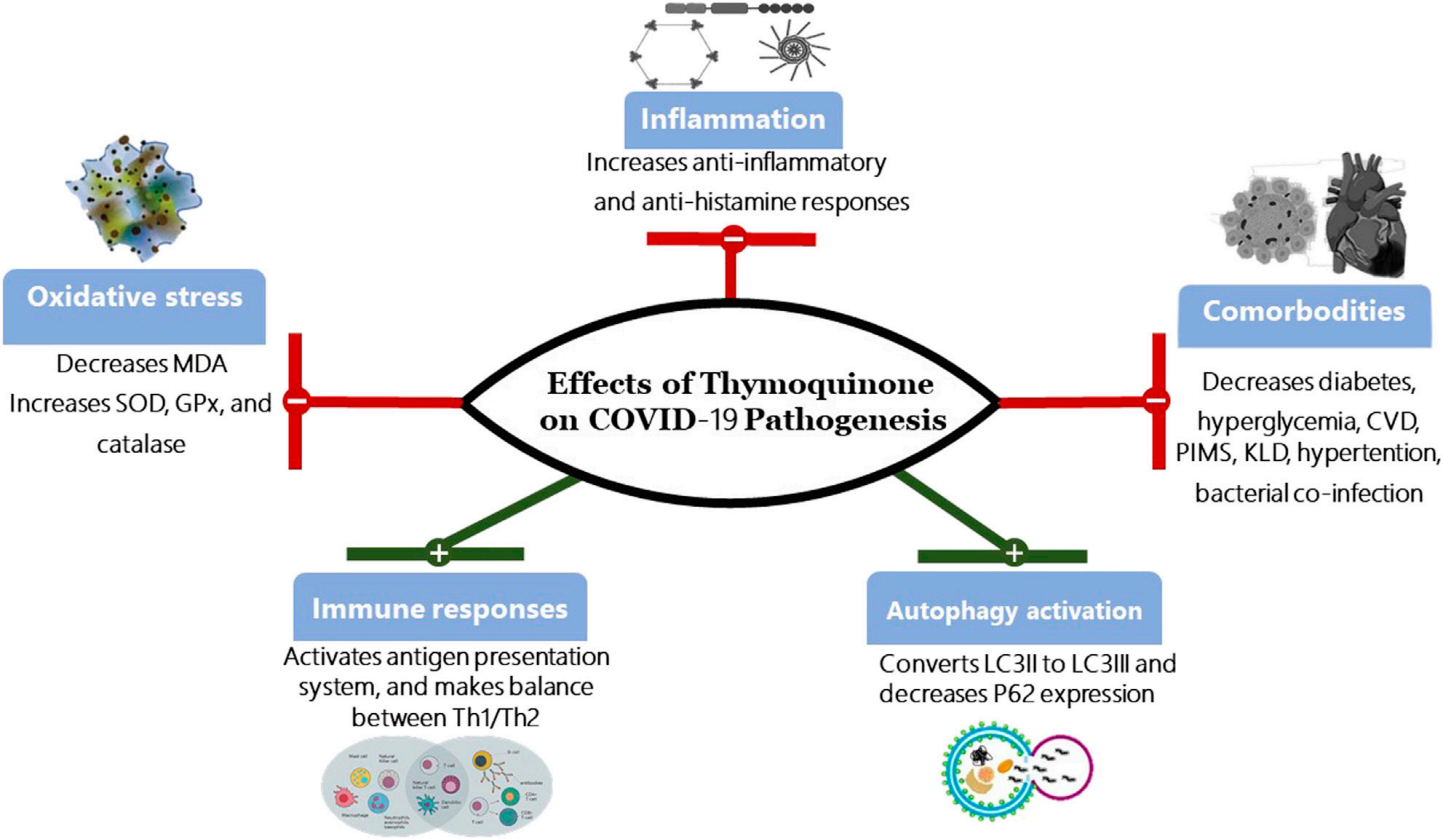
Effects of *Thymoquinone* on COVID-19 Pathogenesis. SOD, Superoxide dismutase; GPx, Glutathione peroxidase; MDA, Malonaldehyde Th1, type I helper T lymphocytes; Th2, type II helper T cells; CVD, Cardiovascular disease; PIMS, Paediatric Inflammatory Multisystem Syndrome; KLD, Kawasaki-like diseases; LC3, Microtubule-associated protein 1A/1B-light chain 3; P62, protein 62.

Interestingly, one study has demonstrated that extracts from multiple plants or their scientific extraction, including *Thymoquinone* (*Nigella sativa*), Anthemis hyalina, and Citrus sinensismay influence the outcomes of coronavirus infections. All three extracts showed possible therapeutic benefit, with Anthemis hyaline, having the most effect (Ulasli et al., 2014). A recent study (under pre-print review) has shown that compounds, other than *Thymoquinone*, extracted from *Nigella sativa* regulate molecular docking (Bouchentouf and Noureddine, 2020). Molecular docking is promising in silico method for screening diverse drugs for their antiviral potential by comparing their binding affinities to various viral or host cell receptor proteins. Various viral proteins involved in viral entry, such as spike proteins, and replication, such as viral proteases, are molecular targets of SARS-CoV-2 (Senger et al., 2020). Additionally, a double-blind randomized controlled trial discovered that *Nigella sativa* extracts reduced inflammatory cytokine response in a patient with rheumatoid arthritis (Hadi et al., 2016). These results are promising in the case of COVID-19 due to the fact that infected patients are in a state of chronic inflammation and at risk of developing cytokine release syndrome. These observations were suggestive of a potential role for *Thymoquinone* in the treatment of COVID-19 Figure 2.

Abdel-Fattah et al. (2000) found that since *Thymoquinone* has antinociceptive effects by indirectly activating the supraspinal μ1-and κ-opioid receptor subtypes, it may prevent SARS-CoV-2 entrance into pneumocytes *via* ACE2. Multiple investigations have found that opioid receptors and ACE have overlapping inhibitory chemicals, for example, Rahman (2020) speculated that *Thymoquinone* might also block ACE2. Takai et al. (1996) additionally proposed that brain endogenous angiotensin II, by its antagonistic interaction with the endogenous opioid system, was implicated in central nociceptive pathways. Furthermore, Lantz et al. (1991) showed that opioid-active peptides, such as hemorphins, have an inhibitory effect on ACE. The above line of evidence suggests that opioid receptors and ACE share similar inhibitory molecules and as such, in publication, Rahman indicated that *Thymoquinone* may also block ACE2 (Rahman, 2020).

In a collaborative research project, Codex Bio Labs tested black seed oil and *Thymoquinone* for their effect on viral entry and viral protein translation using Codex’s Murine Leukemia Virus (MLV) particles pseudotyped (PP) with the SARS-CoV-2 Spike protein (unpublished data). Various combinations/concentrations of black seed oil and *Thymoquinone* were tested against SARS-CoV-2 MLV pseudovirus particles (pp) by assessing Luciferase activities measured with a Firefly Luciferase Assay Kit (CB- 80552-010, Codex BioSolutions Inc.). It was observed that *Thymoquinone* seemed to block viral infection. However, at high concentrations *Thymoquinone* caused cell death indicating cytotoxic effects. To confirm this result, cell growth assays were performed in the presence of *Thymoquinone* with Codex’s EnerCount cell growth assay kit which measures ATP levels inside the cells.

Similarly, in a seropositive HIV infected patient treated with *Thymoquinone* (10 ml twice/day for 6 months), Onifade et al. (2013) demonstrated a decrease in viral load to an undetectable level within 3 months, an increase in CD4 count, relief of symptoms, and a sustained sero-reversion following COVID-19 therapy. Another investigation on a seropositive HIV-infected woman who received *Thymoquinone* (*Nigella sativa*) and honey treatment (10 ml thrice/day for 1 year) demonstrated prolonged sero-reversion, which the author attributed to *Thymoquinone’s* possible virucidal effect (Onifade et al., 2015).

Akhtar and Riffat (1991) demonstrated the efficacy of a single oral administration of *Thymoquinone* (*Nigella sativa*) as powdered seeds and ethanolic extract (40 mg/kg body weight) in reducing the percentage of fecal eggs per Gram in children who were infected with cestodes.

In a study conducted on Hepatitis C (HCV) patients, Abdel-Moneim et al. were able to demonstrate that extracts of *Nigella sativa* (*Thymoquinone*) and *Zingiber officinale*, alone and together (500 mg of *Nigella sativa* and/or Zingiber officinale twice daily for 1 month), improved liver function and decreased viral load in the HCV patients (Onifade et al., 2013). Decreased viral load and improved liver function were similarly reported in another study by Barakat et al. (2013) where HCV patients received capsules of *Nigella sativa* oil (450 mg) three times a day over 3 months. Furthermore, *Thymoquinone* has been studied for benefits other than anti-inflammatory effects which are beyond the scope of this review (Mostofa et al., 2017; Mohammed and Islam, 2018; Shanmugam et al., 2018; Jehan et al., 2020; Leong et al., 2021; Salehi et al., 2021).

## Thymoquinone Studies in Covid-19 Patients

### Clinical Studies

In an investigator-initiated, 313 COVID-19 positive patients were divided into two groups: mild to moderate (cough, fever, sore throat, nasal congestion, malaise and/or shortness of breath) and severe (fever and/or cough along with pneumonia, severe dyspnea, respiratory distress, tachypnea (>30 breaths/min or hypoxia (SpO2 <90% on room air) however, this was conducted as open-label-placebo and randomized controlled trial, 210 and 103 patients were allocated to the mild/moderate and severe groups, respectively, using the clinical care criteria for COVID-19 implemented by Pakistan’s Ministry of National Health Services (Ashraf et al., 2020). Within each of the two groups, the patients were randomly allocated to the treatment group (which received honey + *Thymoquinone* (*Nigella sativa*) [HNS]) or the control group (which received no therapy) (receiving empty capsules). Honey (1 g) and *Nigella sativa* seeds (80 mg) per kg body weight were given orally in 2-3 split doses daily for up to 13 days in the HNS group, whereas the control group got a placebo (empty capsules). The primary outcomes were viral elimination (no RT-PCR for SARS-CoV-2 RNA), clinical symptom relief, and a reduction in Clinical Grading Score (CGS) on day 6. Fever decrease (day 4), C-Reactive protein CRP levels (day 6), the intensity of symptoms (day 8), CGS score (day 10), and death on day 30 were all secondary outcomes. HNS aided with symptom relief and viral clearance, as well as lowering mortality in individuals with moderate and severe illness, according to the findings. COVID-19 symptoms were shown to be relieved earlier in the HNS groups than in the control groups: 4 versus 7 days for moderate patients and 6 versus 13 days for severe disease patients. For both moderate and severe cases, viral elimination (being negative for the SARS-CoV-2 RT-PCR test) occurred 4 days sooner in the HNS group. On day 4, there was a considerable decrease in the severity of fever in the severe patients (OR: 0.21; 95% CI: 0.09–0.46; *p* = 0.0001). On day 6, C- reactive proteins (CRP) levels in both HNS groups reduced dramatically (*p* 0.0001) when compared to their respective control groups. On day 8, 98.13% of patients in HNS-treated mild cases were asymptomatic, compared to 56.31% in the control group (OR: 0.009; 95% CI: 0.001–0.08; *p* < 0.0001). More patients in the HNS group were asymptomatic in severe instances, whereas more in the control arm experienced mild symptoms (median) (OR: 0.1; 95% CI: 0.04–0.24). On day 10, 96.26% of moderate case-patients with HNS had fully resumed regular activities, compared to 68.93% in the control group (OR: 0.07; 95% CI: 0.02–0.21). The median CGS at day 10 for the severe group demonstrated that HNS treated patients returned to normal activities, whereas control patients remained hospitalized and required oxygen treatment (OR:0.05; 95% CI: 0.02–0.15). Morality after 30 days was 18.87% in the control group and 4% with HNS treatment (OR: 0.18 95% CI: 0.02–0.92) (Ashraf et al., 2020).

### Non-Clinical Studies

A molecular docking and molecular dynamics stimulation study conducted by Elfiky (2021) tested the effect of natural products against the HSPA5 substrate-binding domain. The results showed that active components in cinnamon and seeds of *Nigella sativa* may tightly bind to cell-surface HSPA5 (one of the host cell receptors recognized by the viral spike protein) and could be successful in hindering SARS-CoV-2 spike recognition and attachment.

In an, *in vitro* model of rheumatoid arthritis, Vaillancourt et al. (2011) illustrated that *Thymoquinone* significantly decreases lipopolysaccharide (LPS) -induced proinflammatory cytokines such as interleukin1beta (IL-1β), tumor necrosis factor-alpha (TNF-α), metalloproteinase-13 (MMP-13), COX-2, and prostaglandin E2.

Similarly, Gholamnezhad et al. (2019) reported an anti-inflammatory effect of *Thymoquinone* in allergic lung inflammation. There was a *Thymoquinone*-associated decrease in IL-4, IL-5, and IL-13, but an increase in IFN-γ in BALF and lung homogenates.

As part of an *in-vitro* study, Cobourne-Duval et al. compared LPS/IFNγ-activated BV-2 microglial cells (immortalized murine microglial cell line) with and without *Thymoquinone* treatment in a quantitative proteomic study. The following inflammatory cytokines had considerably increased protein expression in LPS/IFNγ-activated BV-2 cells compared to controls: IL-2 (127%), IL-4 (151%), IL-6 (670%), IL-10 (133%), and IL-17a (127%). When comparing the protein expression levels of the same inflammatory cytokines in *Thymoquinone* treated LPS/IFNγ-activated cells to the protein expression levels in activated cells without *Thymoquinone* treatment, the protein expression levels in *Thymoquinone* treated LPS/IFN-activated cells were significantly reduced (*p* < 0.0001). IL-2, IL-4, IL-6, IL-10, and IL-17a levels were reduced by 38 percent, 19 percent, 83 percent, 23 percent, and 29 percent, respectively, when compared to controls (Cobourne-Duval et al., 2018). Additional findings of the study showed that *Thymoquinone* significantly inhibited the production of various inflammatory cytokines in LPS/IFNγ stimulated BV-2 microglial cells, displaying an inhibitory impact on the expression of several interleukins such as IL-2, IL-4, IL-6, IL-10, and IL-17a (Cobourne-Duval et al., 2018).

## Discussion

In pre-clinical studies, *Thymoquinone* has been shown to possess anti-inflammatory properties as well as anti-corona virus properties by blocking viral entry. The acute and sub-acute toxicity of *Thymoquinone* has been examined in various *in-vitro* and *in-vivo* experiments. *Thymoquinone/Nigella sativa* has been studied extensively over many years and has been found to be relatively safe, with very few side effects despite the low level of toxicity that the seed extract and its constituent’s exhibit (Abukhader, 2012; Ong et al., 2016). Furthermore, *Black seed* (*Black Cumin* or *Nigella sativa*) has been categorized by the FDA under spices and other natural seasonings/flavorings that are generally recognized as safe for their intended use (409 of the Act Title 21, Chapter I, Subchapter B, Sec. 182.10 Spices and other natural seasonings and flavorings).

A review of the literature on the therapeutic uses of *Thymoquinone/Nigella sativa* shows some promising results but remains inconclusive. There is a scarcity of studies investigating clinical efficacy, especially at higher doses. Furthermore, any results derived from preclinical studies are confounded by the use of varied extracts thus introducing heterogeneity in the product being tested. More rigorous pre-clinical and clinical research studies need to be conducted before *Thymoquinone/Nigella sativa* can be routinely used as an effective complementary or alternative treatment.

In addition to efficacy, alternative medicine must also satisfy safety criteria. For instance, it is a misconception that these substances are always healthy, since, in addition to possible intrinsic adverse effects, marketed preparations may also have additives that can increase the risk of negative side effects. For example, *Thymoquinone* was found to inhibit CYP enzymes, particularly CYP29C, which may lead to possible interactions (Albassam et al., 2018).

Importantly, the regulatory processes governing complementary and alternative medicine preparations are not as strict as for other pharmaceuticals. The devastating health effects of the COVID-19 pandemic have led to the use of a variety of non-evidence-based treatments that are yet to be validated by large, randomized control trials.

## Conclusion

Although multiple studies indicate promising beneficial effects of *Thymoquinone* in the treatment of various diseases, the current body of research is limited in terms of its scope, quality, and quantity. Physicians are discouraged from recommending the use of marketed supplements of natural products, including *Thymoquinone,* for COVID-19. Given the numerous suggested positive effects of *Thymoquinone,* including its anti-inflammatory, additional research is required to confirm these promising benefits or refute the suggested benefits.
